# CpG island hypermethylation go circular (RNA)

**DOI:** 10.18632/oncotarget.26074

**Published:** 2018-09-04

**Authors:** Mihnea P. Dragomir, George A. Calin

**Affiliations:** George A. Calin: Department of Experimental Therapeutics, The University of Texas MD Anderson Cancer Center, Houston, TX, United States of America

**Keywords:** circular RNA, CpG island, DNA methylation, epigenetics

In recent years the ever-expanding category of non-coding RNAs (ncRNAs), was joined by a new class of transcripts, circular RNAs (circRNAs). CircRNAs are highly conserved, abundant, stable, and are located in the cytoplasm – all these being indirect arguments of their functionality [1, 2]. CircRNAs are RNA molecules generated from pre-messenger RNAs (pre-mRNAs), by a process termed back-splicing, leading to the formation of circular uninterrupted loops, in which the 3’ and 5’ ends are joined together. The biogenesis of circRNAs from pre-mRNA is a well described mechanism; the back-splicing process can be induced by exon-skipping, intron pairing and RNA binding proteins [3]. It was found that many circRNAs work as sponges for microRNAs (miRNAs), blocking their downstream targeting activity [4]. In numerous types of cancer, circRNAs are up- or down-regulated, having oncogenic or tumor suppressor functions, respectively [4]. The efficiency of the back-splicing mechanism can only partially explain the altered levels of circRNAs observed in cancer. What exactly determines the deregulated expression of circRNAs in malignant disease remained, until recently, an almost unexplored field.

Starting from the observation that the levels of coding and other ncRNA transcripts, such as miRNAs and long-non-coding RNAs, are down-regulated in different cancers, not only by deletions and amplifications, but also by epigenetic promotor CpG island hypermethylation [5, 6], Ferreira and colleagues hypothesized that the expression of circRNAs could also be epigenetically silenced [7].

In a well-designed approach, the authors compared, using circRNA microarray, the levels of circular transcripts between HCT116 and a double knockout for DNA methyltransferase 1 and DNA methyltransferase 3B HCT116 (DKO). Additionally, they normalized the results to normal colon tissue. They discovered 18 circRNAs that are overexpressed in normal colon tissue and DKO, but not in wild type HCT116. Furthermore, they used a methylation microarray, to determine which of the 18 circRNAs differently expressed are subject to CpG island hypermethylation and found that only 6 circRNAs belonging to 4 host genes are down-regulated because of promotor CpG silencing. In order to check the function, the authors predicted the miRNAs complementary to the circRNAs down-regulated in HCT116, and found that 3 of the circRNAs sponge at least one miRNA. The most interesting candidate from the six circRNAs was circ104557 which belongs to the host protein coding gene TUSC3 (tumor suppressor candidate 3) that encodes a protein with well-known tumor suppressor role [8]. The *in vivo* xenograft mouse model proved that forced overexpression of circ104557 decreases the tumorigencity of HCT116.

Finally, to prove that their findings are not restricted to a single cell line, the authors analyzed a collection of 1001 human cancer cell lines from the Sanger institute and showed that for the vast majority of colorectal cancer (CRC) cell lines the CpG island of TUSC3 is hypermethylated and correspondingly the expression of TUSC3 is down-regulated in these cells. Using the TCGA patient samples they confirmed the *in vitro* data, the CpG island of TUSC3 is hypermethylated and the level of the transcript is lower in methylated compared to unmethylated samples [7].

**Figure 1:**
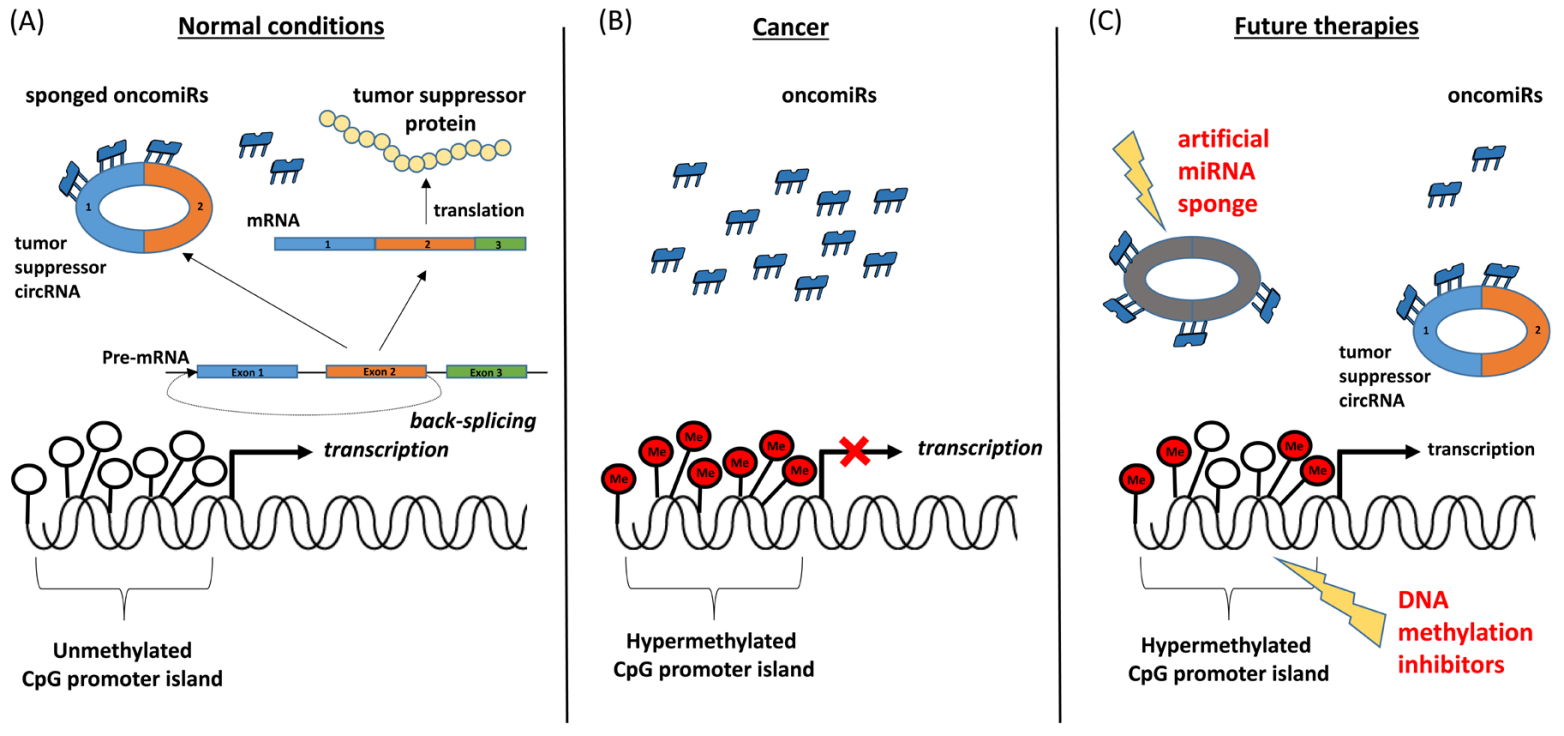
CpG promoter island hypermethylation silences tumor suppressor circRNA in cancer cells **A.** In normal conditions the CpG promoter island of tumor suppressor genes is not methylated and transcription is active. Simultaneously the linear transcript that encodes for a tumor suppressor protein and the circular transcript that sponges oncomiRs play anti-tumorigenic roles. **B.** In cancer, because of the hypermethylation of the CpG promoter, both the expressions of circRNAs and tumor suppressor proteins are silenced, these events leading to the overexpression of oncomiRs and cancer progression. **C.** Based on the date from this published paper, we envision two possible therapeutic approaches: (1) the manipulation of methylation levels by therapeutic agents that will restore the circRNA levels and consequently down-regulate the interactor miRNAs, and (2) the use of artificial miRNA sponges, with structure similar to the natural occurring tumor suppressor circRNAs, which will inhibit the oncomiRs activity.

The interesting results of this well performed study have important translational potential and open new unexpected therapeutic avenues. In CRC almost all cell lines, independent of microsatellite status, are hypermethylated in the promotor region of TUSC3 and most of the analyzed patients confirm this data, this being the premises of an ideal therapy: an alteration present in all CRC patients, unrelated to stage and absent in normal tissue. Hence, reversing methylation levels by therapeutic agents could block the epigenetic silencing of TUSC3 and consequently restore its corresponding tumor suppressors circular and linear transcripts. An alternative therapeutic strategy would be to tackle the end of the molecular pathogenic chain, by using miRNA sponges, which would substitute the natural occurring circRNAs that inhibit the tumorigenic miRNAs (Figure 1) [9].

CircRNAs were recently discovered in different bodily fluids and are promising cancer biomarkers, which could lead to the early detection of cancer patients [10]. Therefore, it is interesting to expand the research presented in this study, and check if the 6 reported circRNAs are circulating in bodily fluids and if their down-regulation is cancer specific or related to phenotypic characteristics of tumors and, most importantly, to patients survival and/or response to therapy.

The study of Ferreira et al. reunites multiple pieces of the cancer puzzle, helping us better understand the complex and intricate mechanism of oncogenesis. It is clear that cancer pathogeny is more complex than mutations or expression variations of protein tumor suppressor genes and oncogenes, and the paper by Ferreira and colleagues is a strong argument in this view. There is a wide network composed of DNA epigenetic changes that control the expression of coding and ncRNA; elements of the ncRNA regulate one another (e.g. circRNAs sponge miRNAs) and the ncRNA regulates the coding genes at all levels (including the DNA methylation enzymes). It seems to be a highly orchestrated vicious circle and the big questions are where does it all start and how can such complex pathologic states be kept under control therapeutically?
